# An Overview of Polymeric Nanoplatforms to Deliver Veterinary Antimicrobials

**DOI:** 10.3390/nano14040341

**Published:** 2024-02-09

**Authors:** Yaxin Zhou, Lihua Guo, Guonian Dai, Bing Li, Yubin Bai, Weiwei Wang, Shulin Chen, Jiyu Zhang

**Affiliations:** 1 Key Laboratory of New Animal Drug Project of Gansu Province, Lanzhou 730050, China; zhouyaxin@caas.cn (Y.Z.); dai940910@163.com (G.D.); pharm2005bl@126.com (B.L.); baiyb1011@163.com (Y.B.); weiweiwang1990@163.com (W.W.); 2 Key Laboratory of Veterinary Pharmaceutical Development, Ministry of Agriculture, Lanzhou 730050, China; 3 Lanzhou Institute of Husbandry and Pharmaceutical Sciences, Chinese Academy of Agricultural Sciences, Lanzhou 730050, China; 4 Shenniu Pharmaceutical Co., Ltd., Dezhou 253034, China; lihuaguo1980@sina.com; 5 College of Veterinary Medicine, Northwest A & F University, Yangling 712100, China

**Keywords:** polymeric nanoparticles, antibiotic delivery, natural polymers, synthetic polymers, veterinary drugs

## Abstract

There is an urgent need to find new solutions for the global dilemma of increasing antibiotic resistance in humans and animals. Modifying the performance of existing antibiotics using the nanocarrier drug delivery system (DDS) is a good option considering economic costs, labor costs, and time investment compared to the development of new antibiotics. Numerous studies on nanomedicine carriers that can be used for humans are available in the literature, but relatively few studies have been reported specifically for veterinary pharmaceutical products. Polymer-based nano-DDS are becoming a research hotspot in the pharmaceutical industry owing to their advantages, such as stability and modifiability. This review presents current research progress on polymer-based nanodelivery systems for veterinary antimicrobial drugs, focusing on the role of polymeric materials in enhancing drug performance. The use of polymer-based nanoformulations improves treatment compliance in livestock and companion animals, thereby reducing the workload of managers. Although promising advances have been made, many obstacles remain to be addressed before nanoformulations can be used in a clinical setting. Some crucial issues currently facing this field, including toxicity, quality control, and mass production, are discussed in this review. With the continuous optimization of nanotechnology, polymer-based DDS has shown its potential in reducing antibiotic resistance to veterinary medicines.

## 1. Introduction

Over the last century, the decrease in morbidity and mortality from infectious diseases has mainly been attributed to the use of antibiotics [1]. Recently, however, the World Health Organization revealed that the threat of antibiotic resistance has reached unprecedented levels because of the prevalence and increase in antibiotic resistance in bacteria worldwide [2]. The emergence of bacterial resistance means that the therapeutic effects of traditional antibiotic formulations are far from satisfactory. We are thus faced with a situation either to figure out new antibiotics or to develop new delivery techniques to enhance the efficiency of the current ones [3,4]. Developing a new generation of antibiotics is the most practical strategy; therefore, various antibiotic substitutes, such as antimicrobial peptides, antibacterial vaccines, probiotics, phages, and other products, have become current research hotspots [5]. Unfortunately, there is no guarantee that the development of new antimicrobials can keep up with the rapid and frequent development of resistance by microbial pathogens. The research for a new antibiotic typically costs more than USD 500 million and requires 10 years to develop, while widespread resistance emerges in less than 3 years [6]. Additionally, research funding for developing new antibiotics is significantly insufficient. The European public sector investment funds only invest approximately EUR 430 million annually in antibiotic development, and just USD 1 billion was provided from the Antimicrobial Resistance (AMR) Action Fund of the Horizon Research and Innovation Program of the European Commission to deal with this issue [7]. In addition, existing alternatives have a large gap with antibiotics in terms of bacterial inhibition and their own stability. For example, antimicrobial peptides are considered highly promising alternatives to antibiotics; however, their conversion into clinical applications is hindered by challenges such as poor in vivo stability, rapid degradation, and susceptibility to inactivation in the physiological environment [8]. Antibacterial vaccines are effective in preventing bacterial infections, but the range of diseases that can be controlled using vaccines is currently limited [9]. Although phage therapy has attracted the interest of scientists owing to its powerful natural killing effect, a primary drawback of using whole phages for infection treatment is the inherent viral nature of the phage [10].

With rapid progress in material science, nanochemistry, and nanobiotechnology, compared to the research and development of new antimicrobial drugs and antibiotic substitutes, a new field using antimicrobial nanomaterials and/or nanocarriers to enhance the existing antimicrobial drugs and reduce the antibiotic resistance effect is showing many advantages, such as being more economical, practical, valuable, and efficient. In the past few decades, the use of nanotechnology in medicine has been widely explored in many fields, especially DDS [11]. DDS emerged in the late 20th century, in which drugs are encapsulated in a carrier material for delivery. DDS has the unique advantage of allowing the controlled release of drugs. As a result, it can improve targeting and bioavailability as well as reduce toxicity and side effects. DDS has been widely used for drug development in humans, and its potential application in preparing veterinary medicines is also significantly recognized [12,13,14]. For antibacterial drugs, the crucial pharmacokinetic properties of antibiotics, including enhanced solubility, controlled release, and site-specific delivery, can be achieved using DDS [15]. Zhang et al. designed a polymer-nanoparticle-modified microrobot to deliver ciprofloxacin for the targeted treatment of acute bacterial pneumonia. The microrobot was retained in the lungs for more than 2 days after intratracheal drug administration to mice and effectively reduced the abundance of *Pseudomonas aeruginosa* (*P. aeruginosa*) with no signs of acute toxicity, thereby reducing mortality [16].

**Figure 1:**
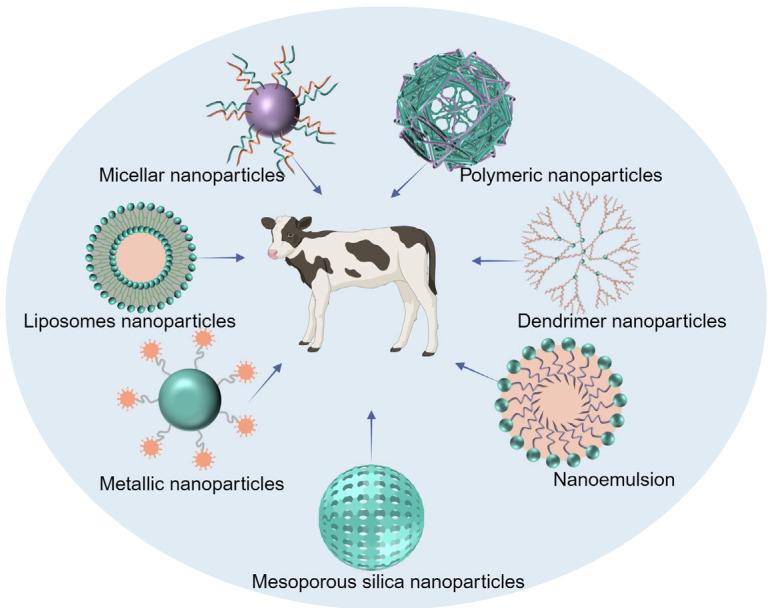
Schematic representation of nanocarriers used for veterinary medicines (the figure was created with BioRender.com).

The use of animals is being increasingly recognized as an important means for human disease research. Thus, research on veterinary medicines provides opportunities for initiative and imaginative design in areas where human pharmaceuticals are not available. In recent years, there has been a growing interest among pharmaceutical companies specializing in the veterinary field to develop DDS specifically for animals [17,18]. For veterinary therapy, the pharmaceutical market primarily focuses on two main groups of animals, namely, livestock and companion animals. The use of DDS for livestock groups offers several key advantages. First, it reduces the workload for veterinarians by minimizing the need for excessive handling. Secondly, it results in a decreased stress response in animals, which ultimately lowers treatment costs. The initial cost is probably higher for developing DDS than for conventional dosage forms; however, the long-term economic benefits outweigh this drawback [19]. For pets, the use of DDS helps improve treatment compliance and increases the ease of administration by owners themselves, as animals are noncompliant [20]. Many kinds of nanocarriers have been investigated, including liposome nanoparticles (NPs), micellar NPs, polymeric NPs, dendrimer NPs, metallic NPs, mesoporous silica NPs (MSNs), and nanoemulsions, to prepare DDS for veterinary medicines [21,22,23,24,25,26,27]. Drugs are loaded into these NPs generally by physical encapsulation, adsorption, dispersion, and coupling. The structures of these nanocarriers are shown in Figure 1. Additionally, the safety of nanomedicine delivery systems used in the veterinary field has received increasing attention because some food-producing animals will enter the food chain and affect human health. Some institutions are already taking corresponding measures to address this issue. In 2021, the US FDA set up the Center for Veterinary Medicine Nanotechnology Programs aimed at studying the safety of nanomedical drugs used in food-producing animals to provide support for the subsequent development of nanomedicines [28].

Owing to their unique nanostructure, antimicrobial NPs display many advantages over conventional antibiotics in terms of increased drug solubility, targeted release, minimized drug resistance, and reduced cost. Various nanosized drug carriers can be utilized to enhance the administration of antibiotics by improving pharmacokinetics and accumulation as well as simultaneously mitigating the adverse effects of antibiotics [29,30]. Like DDS, antimicrobial polymer NPs have received increasing attention in recent years owing to their potential applications in the medical and pharmaceutical fields. Polymeric NPs are stable in the gastrointestinal (GI) tract as the encapsulated drug is protected from low pH, enzymatic degradation, and drug efflux. In addition, the rate of drug release from the polymer system can be altered by adjusting the molecular weight or structure of the polymer [31].

Here, the recent research progress of polymeric nanocarriers for use in delivering veterinary antimicrobial drugs is systematically summarized. In particular, this review focuses on categorizing the literature on the basis of polymeric nanomaterials and highlights the role of polymeric drug delivery vehicles in terms of the performance enhancement of antimicrobial drugs. In addition, the key issues encountered in translating the use of polymeric nanocarriers in clinical applications have also been discussed. This review may help readers develop more effective polymeric nanomaterials that can enhance the activity of existing veterinary antimicrobial drugs as an approach to overcoming the problem of drug-resistant bacterial infections.

## 2. Polymer NPs as Antimicrobial DDS

Polymer NPs have been widely used in nanomedical applications due to their biocompatibility and biodegradability. As of 2018, polymer-based NPs are the most popular class of nanomedicines approved by the Food and Drug Administration (FDA), compared to lipid-based NPs and inorganic NPs [32]. Langer and Folkman created the first polymer-based DDS for macromolecule delivery in 1976; however, initial treatment with polymeric NPs was ineffective because they were rapidly cleared by the reticuloendothelial system after intravenous injection [33]. This limitation was subsequently overcome by the discovery of long-circulating, invisible polymeric NPs in 1994 [34].

Polymeric NPs have several unique properties when used for antimicrobial drug delivery: (1) The preparation of antimicrobial polymeric NPs is cost-effective when compared with the synthesis of antibiotics. (2) The stability of polymeric NPs is reflected in the following two aspects: structural stability, which mainly refers to the ability to withstand harsh conditions during preparation (for example, spray drying and ultrafine milling) and storage, and gastrointestinal stability [35]. (3) Particle characteristics, including size, zeta potential, and drug-release behavior, can be precisely adjusted by changing the polymer length during synthesis. (4) The surface of polymeric NPs usually contains functional groups, and modification of the nanoparticle surface using chemicals, physical reagents, or biomolecules can achieve targeted release. Additionally, ligand modification can enhance the permeability and oxidative stress levels of nanomaterials. A glutathione-responsive polymer core modified with hyaluronic acid to form dual cascade-responsive NPs (sNP@G/IR) has been reported, which can sequentially trigger deep penetration, kill bacteria in tumors, and control the targeted release of the drug [36].

**Figure 2:**
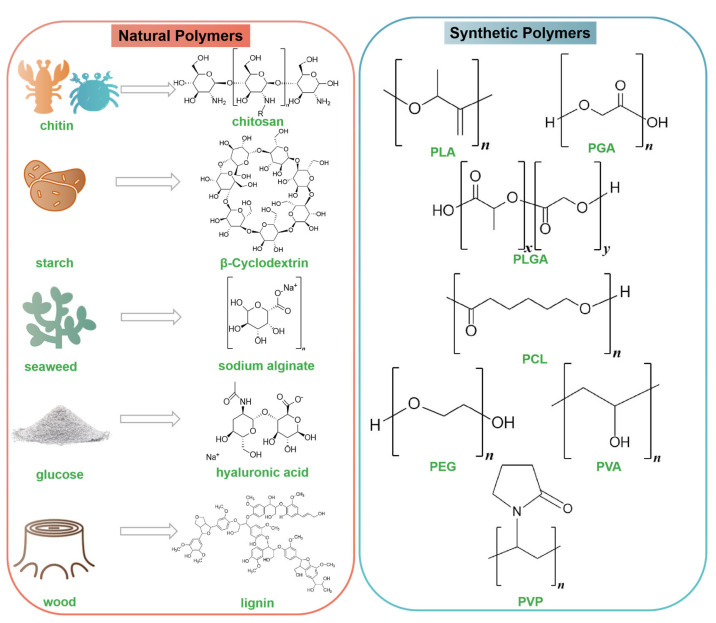
Types of polymeric NPs used for antimicrobial medicines.

So far, polymeric NPs used in antimicrobial medicines can be divided into natural polymers and synthetic polymers. Their chemical structures are listed in Figure 2. Natural polymers are macromolecules that are obtained directly from nature or produced by living organisms. There are reports on chitosan (CS), cyclodextrin (CD), sodium alginate, hyaluronic acid (HA), carboxymethyl cellulose (CMC), hydroxypropyl methylcellulose (HPMC), and lignin as natural polymers [37,38,39,40,41,42,43]. Natural polymers are biocompatible, biodegradable, easily accessible, and easily modified; in addition, numerous natural polymers are known to have specific interactions with organs or cells [44]. Some natural polymers display a higher affinity for cellular receptors, as evidenced by their ability to modulate cellular processes, including adhesion, proliferation, and migration, which indicates the potential for designing more specific and targeted DDS [45]. Another type of antimicrobial polymer NPs are synthetic polymers, which are a promising approach to curbing antibiotic abuse. The most commonly used synthetic polymers in the development of antimicrobial DDS are poly(lactic acid) (PLA), poly(glycolic acid) (PGA), poly(lactic-co-glycolic acid) (PLGA), poly(-caprolactone) (PCL), polyethylene glycol (PEG), poly(vinyl alcohol) (PVA), poly(acrylic acid) (PAA), and poly(vinylpyrrolidone) (PVP) [46,47,48,49,50,51]. These polymer materials have attracted increasing interest owing to their desirable characteristics. First, although these polymers are synthetic, they can degrade to monomers and oligomers in vivo and can be further eliminated by normal metabolism. PLA is a biomaterial that is safe for the environment as well as the human body. Its breakdown products, H_2_O and CO_2_, are also safe [52]. PLGA is an FDA-approved biodegradable polymer; its hydrolysis products are lactic acid and glycolic acid, two monomers that are readily metabolized by the Krebs cycle of the host organism [53]. Second, synthetic polymers can improve the permeability of biofilms. Functionally modified and loaded PEG NPs can easily enter biological membranes by electrostatic interactions, insertion, and disruption [54]. Third, the copolymerization of two monomers or polymers can yield products with several useful properties. Moreover, adjusting the ratio of each component of the copolymer can change the degradation characteristics and drug-release rate. For example, the GA/LA ratio determines the degradation time and stability of PLGA. Lower GA content leads to rapid degradation [55].

As both natural and synthetic polymers are modifiable, polymers can be combined with each other. In addition, polymers can also be combined with different types of NPs (such as liposomes, metal NPs, and mesoporous silica NPs). This hybrid type of nanocarrier can superimpose the advantages of each part of the carrier to further improve the ability to transport antibiotics. In an interesting study, host molecules containing β-CD and MSNs spontaneously formed a supramolecular assembly with guest molecules composed of magnetic core nanoparticles (MagNPs) and MSNs [56]. The delivery platform could simultaneously release the macromolecular antimicrobial peptide melittin and the low-molecular antibiotic ofloxacin. In vivo models showed that the supramolecular nanoplatform not only eradicated pathogenic biofilms but also had no obvious toxicity to mammalian cells. This type of DDS provides a new approach to transporting multiple drugs that have different physical and chemical properties.

## 3. Polymeric NPs for the Delivery of Veterinary Antimicrobial Medicines

**Figure 3:**
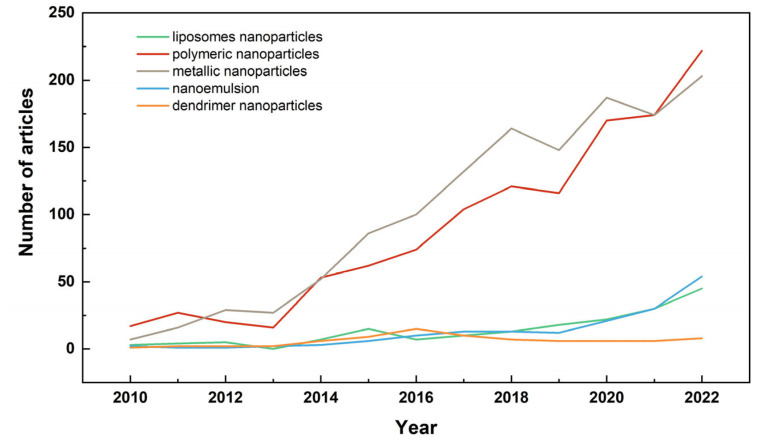
The trend line obtained by PubMed using the query terms “veterinary” and five nanocarriers (“liposomes NPs”, “polymeric NPs”, “metallic NPs”, “nanoemulsion”, and “dendrimer NPs”) over the past decade or so.

Over the past decade, many studies related to various nanocarriers for use in veterinary pharmacotherapy have been reported. The growth trend is shown in Figure 3, and the data were obtained from PubMed. As polymeric NPs have several attractive characteristics for veterinary science applications, including a high specific surface area, a high charge capacity, and the possibility of loading lipophilic and hydrophilic drugs, their research in the veterinary field has been rapidly increasing in recent years. As a result, the number of publications for polymeric NPs is higher than that of other nanocarriers. The following section describes the application of polymeric nanocarriers in veterinary antimicrobial drugs according to different material types.

### 3.1. Natural Polymeric Nanocarrier Delivery Systems

Table 1 summarizes the applications of natural polymer nanocarriers for the delivery of veterinary antimicrobials. CS is the main component of crustacean shells or fungal cell walls. It is a highly biocompatible and biodegradable polymer and has been declared “generally recognized as safe” by the US FDA [57,58]. CS has the function of breaking tight junctions of epithelial cells and increasing permeability, which can enhance the drug transport path. CS itself also has broad-spectrum antibacterial activity and is thus widely used in antibacterial DDS [59,60]. For example, ciprofloxacin-loaded CS NPs (CPX-CS NPs) prepared by Preeti et al. using the ionic gelation method were effective in the treatment of bovine mastitis. The minimum inhibition concentration (MIC) of CPX-CS NPs against *Escherichia coli* (*E. coli*) and *Staphylococcus aureus* (*S. aureus*) was found by the authors to be 50% lower than that of ciprofloxacin hydrochloride alone. Thus, the prepared NPs could reduce the dose of the antimicrobial drug. From a cytotoxicity study, CPX-CS NPs were found to have little or no toxicity, and the 50% inhibitory concentration (IC50) was confirmed to be >1 mg/mL [61]. Physical modifications, such as combining two or more polymers, can broaden the applications of CS. Maryam et al. developed antimicrobial nanofiber mats composed of CS and cellulose acetate loaded with erythromycin NPs (Ery-CS NPs/CA) as a dressing to treat infected wounds. The MIC values of Ery-CS NPs/CA were approximately 5 μg/mL against *S. aureus* and 20 and 80 μg/mL against *E. coli* and *P. aeruginosa*, respectively, which were lower than those for erythromycin and CS NPs. It is worth noting that CS NPs also exhibited high bactericidal activity against Gram-positive and Gram-negative bacteria, although they were not as effective as Ery-CS NPs/CA. The authors mentioned that the antibacterial activity of CS NPs was attributable to changes in membrane permeability, leading to membrane rupture and cytoplasmic leakage [62]. However, the exact mechanism by which CS actually exerts its antibacterial effect is still under debate. Some studies support the above hypothesis of the effect of CS on the cell membrane, whereas others hypothesize that the intranuclear binding of CS to the target microbial DNA results in the inhibition of mRNA activity. The third mechanism relies on the chelation of metal ions by the amino groups of CS, and this effect is observed at pH > 6. A final hypothesis is that CS acts as a barrier, preventing nutrients and oxygen from reaching the bacteria [63,64].

Cyclodextrins (CDs) are cyclic oligosaccharides composed of glucose units and are a type of carrier molecule used to improve pharmacokinetic parameters [65]. Recently, Mariana et al. reviewed the application of CDs in veterinary preparations, noting that CDs are frequently used in veterinary medicines and that their greatest advantage is that they can significantly increase the solubility of insoluble drugs [66]. For example, the solubility of solid complexes of norfloxacin and β-CD prepared by physical mixing and kneading methods increased by two and three times, respectively, compared to norfloxacin alone, and the thermal stability of the drug was also improved [67]. Additionally, CD can also be used to mask the bitter taste of drugs and improve their palatability. Enrofloxacin and florfenicol have a bad smell, and animals often refuse to feed when they are given directly or mixed with food. Wei et al. reported that ultrasonic-synthesized γ-CD metal–organic frameworks coated with enrofloxacin and florfenicol resulted in masking capsules that exhibited lower MIC values and longer half-lives than free drugs [68]. It should be noted that although γ-cyclodextrin has a larger cavity and a wider range of host molecules, its high production cost and limited output limit its application and development. Meanwhile, β-cyclodextrin has a moderate cavity, a simple production process, and a low price, making it currently the most widely used option in the veterinary drug market.

**Table 1:** Natural polymeric nanocarriers used for the delivery of veterinary antimicrobials.

Polymer	Encapsulated Antimicrobials	Animal Model	Administration Route	Bacteria	Performance	References
CS	Ciprofloxacin	-	In vitro	*E. coli* and *S. aureus*	Ciprofloxacin-loaded CS NPs could provide controlled drug release that depended on the pH of the surrounding tissues.	[60]
CS and cellulose acetate	Erythromycin	-	Patch (topical)	*E. coli* and *P. aeruginosa*	The patch inhibited the growth of Gram-positive and Gram-negative bacteria while having no cytotoxicity on human dermal fibroblast cells.	[61]
CS	Chlorhexidine and miconazole nitrate	-	In vitro	*S. aureus*, *S. pseudintermedius*, *M. pachydermatis* and *M. canis*	Chitosan-based gel formulations showed viscosity, syringeability, spreadability, and bioadhesion suitable for topical application.	[69]
β-CD	Norfloxacin	-	In vitro	*S. epidermidis*	The antimicrobial activity of the norfloxacin-β-CD complex was better than that of pure norfloxacin.	[67]
γ-CD	Enrofloxacin and florfenicol	-	In vitro	*E. coli* and *S. aureus*	Drug-loaded γ-CD had higher and longer inhibitory activity than free antibiotics.	[68]
HA	Levofloxacin	Mice	Intravenous	*S. aureus*	NO-sensitive HA-based nanomicelles could achieve lung-targeted delivery and the controlled release of levofloxacin.	[70]
HA	Moxifloxacin	Rabbits and rats	Ocular implant	*P. aeruginosa* and *S. aureus*	Following implantation, moxifloxacin delivery was maintained in the anterior chamber of the eye at a concentration sufficient to inhibit bacteria for more than 5 days.	[71]
Sodium alginate, gelatin, and guar gum	Tilmicosin	Mice	Oral administration	*Lawsonia intracellularis*	Composite nanogels showed enhanced therapeutic efficacy against *L. intracellularis* in vitro and in vivo.	[72]
Sodium alginate and HPMC	Cefpodoxime proxetil	Rabbits	Oral administration	*E. coli*, *S. aureus*, *S. pneumoniae* and *Haemophilus influenza*	Cefpodoxime proxetil matrix tablets exhibited potential as candidates for extended-release dosage forms.	[73]
HPMC	Ciprofloxacin hydrochloride	Rabbits	Oral administration	*-*	DDS prepared from HPMC allowed ciprofloxacin tablets to swell and float in the stomach, maintaining drug release.	[74]
Lignin	Enrofloxacin	-	In vitro	*E. coli* O157: H7	Lignin (enrofloxacin) enhanced the prevention of bacterial infections by more than 50% at lower levels of infection compared to enrofloxacin.	[75]

Natural polymer NPs can also help antibiotics improve their selective targeting of pathological sites. In a recent study, levofloxacin (LF) was attached to HA using an o-phenylenediamine linker to create an NO-sensitive nanosystem (HA-NO-LF) [70]. When exposed to NO, HA-NO-LF nanomicelles could enter host cells via CD44-mediated endocytosis and gradually release the drug. The nanomicelles had a more obvious antibacterial effect on *S. aureus* than LF in a mouse model of pneumonia. An important finding here is that HA, as a carrier of antibiotics, can bind to CD44 on the surface of certain cells, thereby increasing the effective dose of the drug at the site of inflammation [76]. HA can also be used as a drug carrier to treat eye diseases owing to its unique viscoelasticity, biocompatibility, and biodegradability. Dong et al. prepared an intraocular implant by combining HA and moxifloxacin (MXF-HA) to effectively prevent postoperative bacterial infections. MXF release after administration was demonstrated in vivo and was found to be sustained in the anterior chamber of the eye at a concentration adequate to inhibit *P. aeruginosa* and *S. aureus* for more than 5 days after implantation. Thus, this sustained release of antibiotics shows potential in a clinical setting [71].

**Figure 4:**
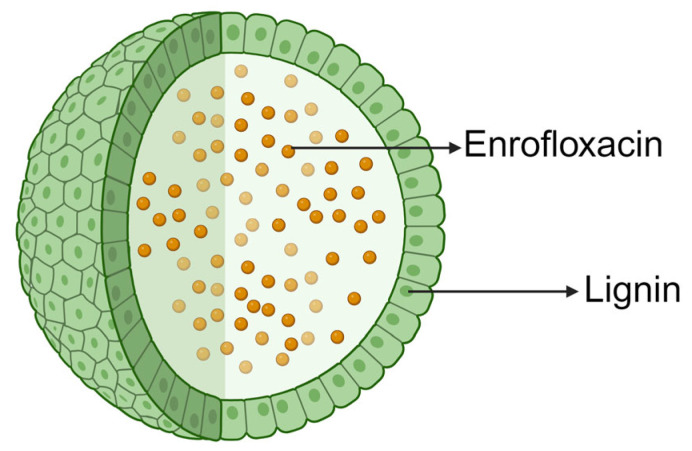
Schematic illustration of lignin-nanocarrier-encapsulated enrofloxacin (figure was created with BioRender.com).

Some natural polymeric materials with adhesive properties can enhance drug delivery and retention in cells. A study has reported a guar gum (GG)-modified, tilmicosin-loaded sodium alginate/gelatin composite nanogel to treat porcine proliferative enteritis caused by *Lawsonia intracellularis*. Tilmicosin nanogels with GG had higher intracellular delivery, retention capacity, and antibacterial activity than those without. Tilmicosin nanogels with GG could completely inhibit the growth of *L. intracellularis* in the intracellular compartments [72]. It is worth noting that this DDS, which is assembled layer-by-layer from a variety of polymeric materials, has obstacles in clinical application. In the long run, the simpler the delivery material utilized, the easier the method of preparation, and the clearer the mechanism of drug delivery, the greater the likelihood that it can be applied. The natural polymer, lignin, has also received increasing attention in recent years. Lignin is the second most highly branched renewable biomacromolecule after cellulose (molar mass typically in the range of 1000–2000 g/mol), which is mainly found in vascular plants, such as herbs and grasses. As lignin has excellent physicochemical properties, including antibacterial and antioxidant effects, as well as biocompatibility and minimal somatic toxicity, it is a suitable candidate for drug delivery applications [77,78]. American scientists Sachin et al. reported that using lignin-nanocarrier-encapsulated enrofloxacin (LNP (Enflx)) could prevent infections caused by pathogenic *E. coli* O157:H7 in intestinal cells. LNP (Enflx) was more effective than Enflx alone by >50% in preventing bacterial infections at low levels of infection [75] (Figure 4). In addition, earlier publications have reported that the antimicrobial effect of lignin itself is species-dependent. In brief, lignin is a natural antimicrobial agent in nonruminants but is less effective in ruminants as lignin may be degraded by rumen microorganisms [79]. Therefore, it is suggested that lignin is more suitable to be used as an antimicrobial drug carrier for nonruminants than ruminants.

### 3.2. Synthetic Polymeric Nanocarrier Delivery Systems

Synthetic polymeric nanocarriers for veterinary antimicrobial drug delivery are summarized in Table 2. The most popular one is PLGA, which is an FDA-approved and well-characterized synthetic polymer carrier for delivering antibacterial drugs. This material has received widespread attention due to the following reasons: (1) Its hydrolyzed monomers, lactic acid and glycolic acid, are endogenous and can be utilized through the triple acid cycle. (2) The copolymerization of two monomers leads to materials with several useful properties. (3) The degradation of PLGA can be achieved by only using water. (4) PLA and PGA are the first polymer materials to be successfully used as sutures in the past two decades. Thus, their safety is well documented [80,81]. Korean scientists have reported the preparation of ciprofloxacin (CIP)-encapsulated PLGA NPs obtained using emulsion solvent evaporation, and the antimicrobial effects against *E. coli* have been evaluated in vitro and in vivo [82]. In drug-release experiments, CIP showed an initial burst release effect for 12 h, followed by sustained release for 2 weeks. From in vitro antibacterial activity tests, CIP-PLGA showed relatively lower antibacterial activity compared to free CIP, which may be caused by its sustained-release properties; however, CIP-PLGA showed excellent efficacy in inhibiting bacterial growth in vivo.

**Figure 5:**
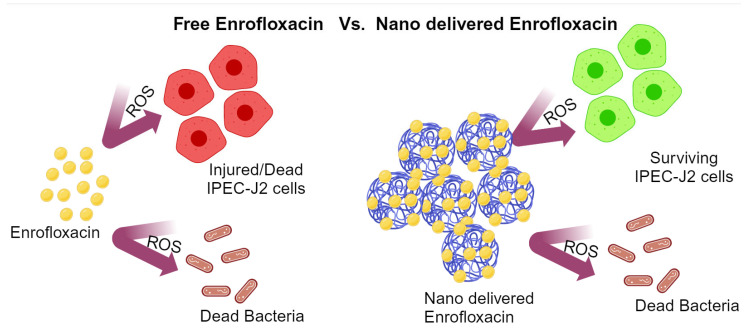
Schematic illustration of free enrofloxacin vs. nanodelivered enrofloxacin (figure was created with BioRender.com).

In another study, the third generation of fluoroquinolone antibiotic enrofloxacin was encapsulated in PLGA in order to solve the problem of excessive reactive oxygen species (ROS) [83] (Figure 5). High levels of ROS can damage some important macromolecules, such as nucleic acids, proteins, and lipids, in bacterial cells and eventually lead to cell death. Thus, controlling the creation of ROS is a key factor in cell apoptosis. Some antibiotics have the effect of causing cell death, which results from a mechanism involving ROS. Enrofloxacin can produce ROS, leading to the death of bacterial and mammalian cells [92,93]. Based on the experimental results, it can be concluded that the use of PLGA can reduce the toxicity of enrofloxacin to cells because of the slow release of the drug substances for as long as 5 days to facilitate the treatment.

PLGA can be used to encapsulate not only individual free drugs but also antibiotic conjugates so as to improve drug bioavailability. In order to improve the efficacy of the antituberculosis drug isoniazid (INH), Kata et al. established an advanced DDS for INH by first coupling the drug molecule to a lipopeptide carrier and then encapsulating the conjugate into PLGA NPs [84]. Compared to the free drug, the conjugate demonstrated better targeting of *M. tuberculosis* in macrophages in a guinea pig model of infection, and the bioavailability of INH was further enhanced by PLGA. Intracellular infections such as *M. tuberculosis* (and other bacteria such as *Salmonella* and *Brucella*) are more difficult to treat than those caused by extracellular bacteria because they evade the host’s immune response, meaning antibiotics often have difficulty penetrating the host cell membrane. The PLGA-encapsulated conjugate system, which specifically kills intracellular *M. tuberculosis*, provides an effective solution to the problem of the intractability of intracellular bacteria.

PCL, another polymer from the polyester family, degrades more slowly than PLA and PGA, making it particularly suitable for long-term DDS [94]. For example, PCL can be used to prepare progesterone-containing intravaginal inserts to control the estrous cycle of livestock. Experimental data showed that PCL can be administered for 7 days in cattle and 14 days in sheep [95]. Related to implantable systems, linezolid-loaded fiber mats were prepared by electrospinning using PCL and PLGA, which were found to be useful in treating and preventing prosthetic infections [85]. An in vivo study of methicillin-resistant *S. aureus*-infected rats with tibial fractures found that the presence of these fiber mats accelerated fracture healing and reduced the antibiotic dose by 37 times compared to that used for conventional treatment.

PEG is chosen as a polymer support owing to its high hydrophilicity to synthesize a water-binding barrier, resulting in low cell adhesion and protein uptake. These properties impart stealth features to PEG, thereby avoiding opsonin binding and further clearance by the immune system [96]. PEG-PLGA NPs loaded with minocycline were prepared by Yao et al. to treat dogs with periodontitis [86]. NPs with an average size of 100 nm, a zeta potential of −24 mV, and an encapsulation efficiency of 46.5% were designed. In vitro drug-release tests revealed higher control of release rates compared to free minocycline (cumulative release of minocycline from the minocycline-containing NPs was approximately 96% after 14 days). It is worth mentioning that these NPs maintained effective drug concentration over a longer period (12 days) than the local periodontal sustained-release drug Periocline^®^.

PVP, a nontoxic mucoadhesive polymer, was used as a DDS in another study aimed at treating periodontal disease. In this study, rapidly dissolving ornidazole-loaded PVP electrospun fibers were prepared using electrospinning to treat gingivitis [90]. As the concentration of PVP increased, the tensile strength, elongation at break, and viscosity of the fiber increased, whereas the conductivity of the fiber decreased. The study findings revealed that 15% PVP resulted in the best formulation. At this concentration, ornidazole could be released from the fiber in 5 min. In vitro diffusion studies showed that fiber formulations diffuse more efficiently than ornidazole-containing gel and solution formulations. In studies determining antimicrobial activity, electrospun ornidazole fibers were found to be as effective as ornidazole solution against *Porphyromonas gingivalis*, which is associated with gingivitis. The main limitation of this report is the lack of further in vivo studies, which restricts the applicability of this PVP DDS due to a lack of data.

### 3.3. Hybrid Polymeric Nanocarrier Delivery Systems

The hybrid polymeric nanocarriers mentioned in this section can be divided into two categories: the first refers to two or more natural and synthetic polymers, and the second is based on the first category but is also doped with other types of nanocarriers, such as liposome NPs or metal oxide NPs. There are many reports in the literature of hybrid polymeric nanocarrier delivery systems for veterinary antimicrobials. The most recent ones are listed in Table 3.

#### 3.3.1. Two or More Polymer-Containing Nanocarrier Delivery Systems

**Figure 6:**
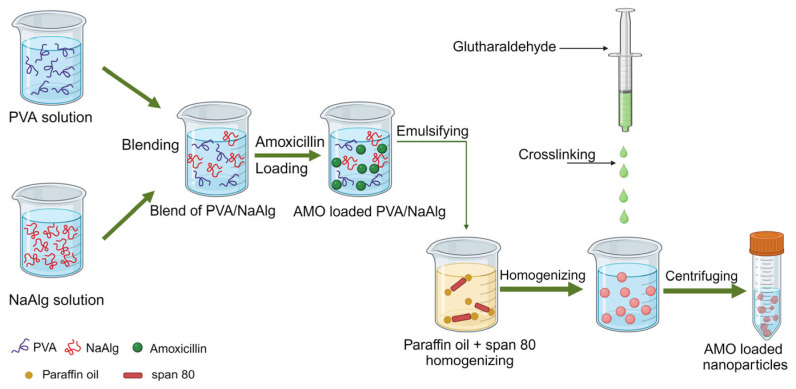
Schematic diagram of the preparation of AMO-loaded nanoparticles (figure was created with BioRender.com).

The first type of hybrid polymeric nanocarrier involves a combination of natural and synthetic polymers. Enes et al. developed a polymer nanosystem consisting of PVA and sodium alginate (NaAlg) for the sustained release of amoxicillin (AMO) [97] (Figure 6). NaAlg was cross-linked with PVA using the polymer blending method in order to solve the problem of the instability of PVA in water [108]. The sample AMO-PVA/NaAlg had an average particle size of 336.3 ± 25.66 to 558.3 ± 31.39 nm and a zeta potential of 41.86 ± 0.55 to 47.3 ± 2.76 mV, and the optimal encapsulation efficiency was 44.51 ± 4.17%. The MIC of this preparation against *S. aureus* was equivalent to that of pure AMO, but its inhibitory performance against *E. coli* was not as good as that of pure AMO, indicating that this preparation did not improve the antibacterial activity of AMO. However, the highlight of this formulation was that it showed a controlled and pH-dependent release of AMO with an initial burst effect. In another report, a combination of PVA and the natural polymer gellan was shown to exhibit an initial burst release of the drug. PVA and gellan were used to prepare ofloxacin nanofibers as a sustained-release system for intragastric use to improve the retention time, bioavailability, pharmacological activity, and therapeutic effect of ofloxacin in the stomach [98]. Results from antimicrobial activity experiments showed that the MICs of ofloxacin nanofibers against *E. coli*, *S. aureus*, *P. aeruginosa*, and *Enterococcus faecalis* were lower than those of pure ofloxacin. In addition, the drug displayed biphasic drug-release characteristics in the gastric mucosa of rats and had certain mucosal adhesion properties.

Polymeric NPs have also been used as an effective strategy to synthesize topical preparations. For example, the main difficulty in treating ocular infections is topical dosing, as multiple daily doses are required to maintain the therapeutic levels of antimicrobial agents. The use of polymeric nanocarriers promotes sustained drug release in the ocular mucosa, maintains a high concentration of nanostructured antimicrobial agents, reduces the number of doses, and minimizes the antibiotic residues in mammalian milk. In a study by Fonseca et al., PCL-coated CS nanocapsules containing cloxacillin were developed to treat infectious bovine keratoconjunctivitis (IBK) caused by *Moraxella bovis* [99]. This was the first study in Brazil using nanoantibiotics for the treatment of clinical IBK. The antimicrobial efficiency of the drug was tested on three naturally infected cows, wherein 1 mL of the preparation (containing 0.32 mg of nanostructured cloxacillin) was administered every 12 h, and no clinical signs were observed after 6 days of treatment. The nanomedicine maintained the MIC in the ocular mucosa for a longer period of time when compared to conventional cloxacillin, although the MIC values were the same. The authors suggested that cows treated with this nanostructured cloxacillin were less likely to have antibiotic residues in their milk and meat. However, more tests and more data are required to support this suggestion. In another study, PVA and HA films were used to simultaneously deliver hydrophilic dexamethasone and lipophilic levofloxacin to ocular tissue in order to treat ocular disease in pigs [100]. The highlight of the study was the finding that the drug-loaded NPs could be targeted to release the drug in specific parts of the eye by adjusting the type of PVA. A film composed of PVA98 and characterized by 2D swelling was suitable for corneal use, whereas a film composed of PVA87 and characterized by 3D swelling was suitable for use in the conjunctival sac.

Polymer-based implants are a promising alternative as they have good biocompatibility (reduced chances of rejection by the body and subsequent inflammation), along with the ability to assist drug release. The delivery forms include transdermal patches and implantable fixtures, among others. Zhu et al. incorporated colistin into a hydrogel composed of a glycol CS and PEG derivative to treat local chronic wound infections [101]. In an in vivo mouse model of “burn” infection, the colistin-loaded hydrogels could kill colistin-resistant *P. aeruginosa* without bringing any toxicity to mice, indicating the potential topical wound-healing properties of the colistin hydrogel. During treatment with implants, bacteria often adhere to the implant surface to form multilayered colonies and eventually grow into a biofilm. The bacteria in the biofilm show higher antibiotic resistance than those in the planktonic state [109]. Recently, there has been an interesting example of using a layer-by-layer self-assembled multilayer film, which not only delivered antibiotics into the infected tissues but also resisted their own bacterial adherence [103]. Using electrostatic force and hydrogen bonding, a multilayer film containing PAA, PVP, and CS at a thickness of about 1400 nm was prepared, on which 153.84 ± 18.64 μg/cm^2^ of gentamicin was loaded. The amount of gentamicin could be precisely adjusted by changing the film thickness. In a rabbit model of *S. aureus* infection, polydimethylsiloxane (PDMS) loaded with multilayer gentamicin films (infection rate 12.5%) exhibited much lower levels of infection than the original PDMS implants (infection rate 75%) after 7 days. Moreover, the gentamicin-loaded multilayer films continuously released drugs to the infected pocket, lasting for 48 h following implantation.

#### 3.3.2. Other Types of Polymer-Based Nanocarrier Delivery Systems

**Figure 7:**
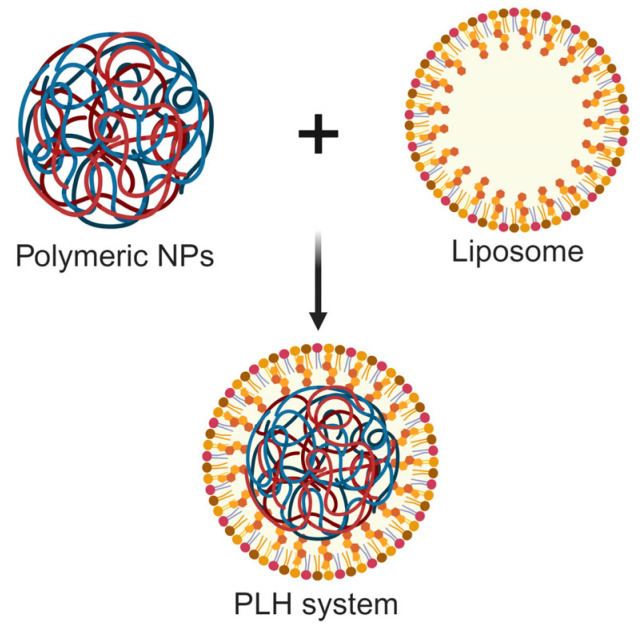
Schematic diagram of the PLH system, which serves as “the best-suited polymeric nanocarriers” for the delivery of veterinary antibacterial drugs (figure was created with BioRender.com).

The polymer-lipid hybrid (PLH) system is a promising one in current DDS. It combines lipid nanocarriers with polymer nanocarriers in order to combine the advantages of lipid solubility and polymer stability to overcome the limitations that affect the applications of conventional lipid preparations [110]. Therefore, it can be used as “the best-suited polymeric nanocarriers” for the delivery of veterinary antibacterial drugs (Figure 7). Recently, Seyed and coworkers evaluated the potential therapeutic efficacy of PEGylated liposomes (PEG-Lip) for delivering the antibiotic nafcillin (NF) in mice [104]. The particle sizes of PEG-Lip-NF and Lip-NF were 239.3 ± 8.5 and 253.0 ± 12.5 nm, respectively; the encapsulation efficiencies were 93.2% and 89.2%, respectively; and the NF release amounts within 30 min were 20% and 24%, respectively. In vitro findings suggested that the MICs of NF, Lip-NF, and PEG-Lip-NF against methicillin-susceptible *S. aureus* (MSSA) 29,213 were 1, 0.5, and 0.25 μg/mL, respectively. In a murine model of bloodstream infection, PEG-Lip-NF reduced the virulence of MSSA bacteremia by 45%, 25%, and 10% compared to the control, NF, and Lip-NF groups, respectively. The results indicated the importance of PEG in PLH systems, in which the smaller size, higher encapsulation capacity, and slower drug release were the main advantages for the higher antimicrobial efficacy of PEG-Lip-NF.

In a follow-up study conducted by the same research group, a PEGylated nanostructured lipid carrier (NLC) was used for testing the oral delivery of trimethoprim/sulfamethoxazole (TMP/SMZ), and the PLH system was evaluated by examining the antimicrobial properties of antibiotics [105]. PEG-TMP/SMZ-NLCs were obtained at a size of 187 ± 9 nm using the melt emulsification method. In vitro, the findings showed that the PEG-TMP/SMZ-NLCs increased the intestinal permeability by 54%, increased the antibacterial efficacy against *S. aureus* by 8-fold, and reduced the toxicity by 2.4-fold. In addition, the antibacterial effect of the nanopreparation increased by three orders of magnitude after oral administration to infected mice when compared to that using TMP/SMZ. The authors speculated that the PLH system (PEG-NLCs) from their study indicated a potential for commercialization of oral formulation preparations. Similar findings were reported by Sajedehsadat et al. [110], who further pointed out that the polymer materials in the PLH system could improve the oral absorption of drugs. Meanwhile, the main mechanisms of this system were stated, which were (1) promoting the mucosal interaction, (2) increasing the residence time of drugs in the GI tract, (3) enhancing drug solubility and inhibiting drug precipitation, and (4) enhancing the biological activity of digestive enzymes, in addition to the “invisible” properties and improved GI stability mentioned in the previous quotations. The authors suggested that the type of polymer material and the mechanism by which the PLH structure affects drug delivery are not yet clear. Moreover, they stated that if the relationship between the PLH structure and drug activity can be elucidated, it could serve as a guide for the subsequent design of PLH formulations.

Another type of DDS was formulated by Kaliyaperumal and coworkers. It was made up of the natural polymers CS and gelatin with the addition of calcium phosphate (CaP) NPs and was used in a wound-healing cream containing chlorhexidine [106]. The best healing result obtained was from the composition with a ratio of 57% gelatin, 14.2% CS, 4.86% CaPs, and 2% chlorhexidine. Gelatin can prevent the loss of the healing cream from the injured sites, CS can maintain moisture on the wound surface, and CaPs can release localized calcium ions on the wound after disintegration at an acidic pH. Results from the in vitro experiments indicated that the antibacterial activity of the chlorhexidine nanocream was three times that of the cream without CS and CaPs. Results from in vivo experiments with mice indicated that the wound size could be reduced by 90–95% after 10 days of using the nanocream. The researchers speculated that this pH-responsive nanocream could be suitable for treating wounds in dogs, and although no supporting data were provided in this regard, the speculation has a theoretical basis. Because the pH of bacterial infection sites (5.5–6.0) is lower than that of other normal tissues in the blood (7.2–7.4), a DDS prepared to take advantage of the diversity of pH at different sites could facilitate the collection of antimicrobial agents in target host tissues.

The composite NPs formed by combining inorganic NPs with organic NPs can significantly increase their antimicrobial properties. Because of that, inorganic NPs have stronger bactericidal effects, and organic NPs have better biocompatibility and lower systemic toxicity [111]. Metal-containing systems, especially metal oxide NPs such as CuO, ZnO, and TiO_2_, have been gradually recognized as a promising class of antimicrobials for combating antibiotic resistance [112]. To harness the broad-spectrum antibacterial effects of ZnO NPs, Behzad et al. prepared a synthetic polymer–natural polymer ZnO nanocomposite based on PVA-CMC nanofibers to deliver erythromycin (EM) [107]. Based on in vitro antimicrobial susceptibility tests, the authors observed that, compared to the nanofibers loaded with EM or ZnO alone, EM-loaded PVA-CMC/ZnO nanocomposite fibers could synergistically inhibit *E. coli* and *S. aureus*. The authors speculated that the combined effect of the physicochemical damage of ZnO NPs and the biomechanism of EM was the main reason for inhibiting bacterial growth. It is noteworthy that PVA-CMC had no antibacterial activity; the combination of the two polymers served only as a drug delivery platform. The antimicrobial activity of PVA-CMC/ZnO was due to the presence of ZnO. As discussed earlier, the fact that metal oxides can be used as a means of drug delivery indicates the dual role of ZnO. However, the toxicity of ZnO is also an important point to pay attention to, as it has been reported that ZnO NPs can cause toxicity in various target organs, including cardiotoxicity, neurotoxicity, hepatorenal toxicity, reproductive toxicity, and immunotoxicity [113].

## 4. Summary and Future Prospects

Over the past few years, several articles on innovative strategies to combat the growth of antibiotic resistance using polymeric NP DDS have been published. This overview aims to summarize the recent research progress on antimicrobial polymeric NPs as new alternatives to traditional antibiotics in the field of veterinary medicine. Polymer-based nanodelivery systems have been playing an important role in the development of new veterinary antimicrobial drugs. DDS can improve bioavailability and reduce side effects compared to conventional antibiotic formulations. There are more advantages for DDS to be used in veterinary medicines as it can reduce the handling of livestock and therefore lower the costs.

The role of polymeric materials in the development of antimicrobials has been an underlying theme in most of the literature surveyed in this review. According to material properties, the polymers are grouped as natural polymers, synthetic polymers, and composite polymers by the authors. Generally speaking, these polymeric materials function as drug delivery carriers, which could enhance intracellular drug delivery and retention. Some of them can also help in the targeted release of drugs. Moreover, some of them, such as CS and lignin, also have inherent antimicrobial activity, which broadens their use in the preparation of antimicrobial drugs. Some polymers, when blended with other materials, can produce extra complementary or synergistic effects, an example of which was discussed in Section 3.3.2.

The use of antimicrobial polymer NPs in veterinary medicine based on current research shows a promising and exciting future. However, there is still a way to go before it has broad clinical applications, and the following key research challenges must first be met.

Carrying out animal model research: The interaction between polymer nanomaterials and cells, tissue accumulation, and biocompatibility during drug delivery should be investigated using in vivo models because in vitro tests may not reflect the true properties of nanomaterials. There are many studies using polymer NPs to design animal-specific antibiotics (enrofloxacin, florfenicol, etc.); however, few of them have used in vivo models. This situation has been seen in this review too.

Exploring the mechanisms of drug delivery: Most studies have focused on evaluating whether polymeric NPs enhance the activity of antimicrobial drugs; however, their mechanisms of drug delivery have not been explored. Once the mechanisms and related influencing factors are clarified, it can help researchers rationally design new iterations of more effective antimicrobial polymeric NPs.

Developing the character techniques for NPs: The size-specific properties of NPs limit the application of certain characterization techniques; thus, there is a need to develop well-defined and innovative physicochemical characterization techniques to better understand the relationship between the structure and properties of polymer NPs, such as nano-time-of-flight secondary ion mass spectrometry and nanoimaging techniques.

Strengthening the quality control of nanomedicines: Currently, most regulatory methods used by international regulatory bodies to evaluate nanoproducts are for non-nanoscale chemicals, and the development of nanodrug supervision methods is also in its infancy [114]. The Center for Drug Evaluation of China issued the “Technical Guidelines for Quality Control Research of Nanomedical Drugs” in August 2021 and the “Technical Guidelines for Quality Control Research of Liposome Drugs” in October 2023 [115,116]. Therefore, it is expected that the development of quality control standards for polymer drugs will come soon.

Conversion from lab to clinic: Another aspect to consider is the possible difficulties in converting or scaling from laboratory work to large-scale production. Challenges in the application of nanomedicine in the veterinary field include the environmental risks of nanomaterials and the high costs incurred during the manufacturing process.

It is worth emphasizing that these challenges are not reflective of the failures of most current antimicrobial polymer nanoparticle DDS but rather illustrate the potential for significant advancements in the field. Overall, the future for veterinary antimicrobial drug applications using polymeric nanoplatforms appears promising. We hope this review inspires new avenues in this exciting field.

## Figures and Tables

**Table 2:** Synthetic polymeric nanocarriers used for the delivery of veterinary antimicrobials.

Polymer	Encapsulated Antimicrobials	Animal Model	Administration Route	Bacteria	Performance	References
PLGA	Ciprofloxacin	Mice	Hypodermic injection	*E. coli*	CIP exhibited the initial burst release effect for 12 h, followed by sustained drug release for 2 weeks.	[82]
PLGA	Enrofloxacin	-	In vitro	*E. coli* and *S. aureus*	Enrofloxacin-loaded NPs led to a reduction in the innate cytotoxicity of the drug while retaining antimicrobial efficacy.	[83]
PLGA	Isoniazid	Guinea pigs	Oral administration	*Mycobacterium tuberculosis* (*M. tuberculosis*)	External clinical signs, histopathology, and other results confirmed that PLGA-loaded NPs had an excellent inhibitory effect on *M. tuberculosis*.	[84]
PCL and PLGA	Linezolid	Rats	Prosthetic implantation	*S. aureus*	Polymer-modified fiber mats were more effective in the treatment and prevention of MRSA-induced prosthetic infections using linezolid at a dose that was 37-fold lower than the normal dose.	[85]
PEG and PLGA	Minocycline	Dogs	Injected into the periodontal pocket	-	Effective drug concentration for 12 days.	[86]
PEG and PLGA	Piperacillin and tazobactam	-	In vitro	*P. aeruginosa*	PLGA-PEG delivered antibiotics into bacterial biofilms and eradicated biofilm formation.	[87]
PLA and PEG	Linezolid	=	In vitro	*MRSA*	PLA-PEG-loaded NPs exhibited inhibitory activity against the proliferation and biofilm of *MRSA*.	[88]
PVP	Ciprofloxacin	-	In vitro	*P. aeruginosa*	PVP foils maintained high drug concentrations in the wound for up to 24 h.	[89]
PVP	Ornidazole	-	In vitro	*Porphyromonas gingivalis*	PVP-modified ornidazole electrospun fibers were found to be a promising drug delivery system for the treatment of oral mucosal infections.	[90]
PCL and PVA	Gentamicin and ciprofloxacin	-	In vitro	*E. coli* and *S. aureus*	PCL/PVA-aspartic acid oligomers could inhibit bacterial growth in vitro for up to 6 days.	[91]

**Table 3:** Hybrid polymeric nanocarriers used for the delivery of veterinary antimicrobials.

Polymer	Encapsulated Antimicrobials	Animal Model	Administration Route	Bacteria	Performance	References
PVA and sodium alginate	Amoxicillin	-	In vitro	*E. coli* and *S. aureus*	The MIC values of the formulation were comparable with the inhibitory activity of pure amoxicillin.	[97]
PVA and gellan	Ofloxacin	Rats	Oral administration	*E. coli*, *Enterococcus faecalis*, *S. aureus*, and *P. aeruginosa*	Compared to the pure drug, gellan/PVA nanofibers showed an initial burst release, followed by the sustained release of ofloxacin for up to 24 h.	[98]
PCL and CS	Cloxacillin	Cows	Ocular	*Moraxella bovis*	Treatment of bovine infectious keratoconjunctivitis with nanostructured cloxacillin for 6 days led to the alleviation of clinical signs after treatment.	[99]
PVA and HA	Levofloxacin	Pigs	Ocular	-	The composite membrane exhibited high loading capacity, controlled drug release, and the ability to deliver dexamethasone and levofloxacin to the cornea and across the sclera to potentially target the posterior segment of the eye.	[100]
PEG and CS	Colistin	Mice	Patch	*P. aeruginosa*	This hydrogel was found to be capable of achieving localized release of active colistin without inducing toxicity in mice.	[101]
PGA and CS	Tetracycline	-	In vitro	*E. coli*	CS/PGA polyelectrolyte multilayers enhanced the inhibitory effect of tetracycline on *E. coli*.	[102]
PAA, PVP, and CS	Gentamicin sulfate	Rabbits	Film-coated implant	*S. aureus*	This composite film could be used as a drug delivery system to achieve sustained release of gentamicin.	[103]
PEG, lecithin, and cholesterol	Nafcillin	Mice	Intravenous	MSSA	Compared to Lip-NF, PEG-Lip-NF showed higher efficacy and reduced the side effects of the drug by 10.5%.	[104]
PEG, lecithin, monostearin, and soybean oil	Trimethoprim and sulfamethoxazole	Mice	Oral administration	*S. aureus*	PEGylated nanocarriers reduced drug toxicity by 2.4 times, increased intestinal permeability by 54%, and increased antibacterial effects in vitro by 8 times.	[105]
CS, gelatin, and CaPs	Chlorhexidine	Dogs	Smearing the wound	*E. coli*, *S. aureus*, and *Salmonella* spp.	A cream containing 0.4 mg/L NPs-CLX showed >70% inhibition of four common bacteria.	[106]
CMC, PVA, and ZnO	Erythromycin	-	In vitro	*E. coli* and *S. aureus*	PVA-CMC/ZnO-EM nanofibers containing the drug had good sustained-release properties and antibacterial activity and were found to be an ideal wound dressing material.	[107]
PGA and MSNs	Chlorhexidine	-	In vitro	*Streptococcus mutans*	The NP system showed high antimicrobial efficacy after incorporation into the dentin-adhesive system.	[47]
